# *Raveniola niedermeyeri* from Iran: redescription and new data on distribution (Araneae, Nemesiidae)

**DOI:** 10.3897/zookeys.57.497

**Published:** 2010-09-21

**Authors:** Sergei Zonstein, Yuri M. Marusik

**Affiliations:** 1 Department of Zoology, The George S. Wise Faculty of Life Sciences, Tel-Aviv University, 69978 Tel-Aviv, Israel; 2 Institute for Biological Problems of the North RAS, Portovaya Str. 18, Magadan, Russia

**Keywords:** Araneae, spiders, Nemesiidae, Raveniola, Iran

## Abstract

Raveniola niedermeyeri (Brignoli, 1972), a poorly known species, is rediagnosed and redescribed from the types and from recently collected material from northern and central regions of Iran. This species differs from its congeners in having the male embolus curved distally, as well as in the unique conformation of the spermathecae. New data on the distribution of Raveniola niedermeyeri in Iran are also provided.

## Introduction

Nemesiidae is the second largest mygalomorph family, containing 350 species (Platnick 2010) and is distributed worldwide. The diplurid Brachythele niedermeyeri was first described by Brignoli (1972) on the basis of a few mygalomorph specimens from Iran collected in the vicinities of Astrabad (now called Gorgan) by Oskar Niedenmeyer prior to World War I . Later, this species (hitherto known only from the type locality) and its closest relatives were transferred to the nemesiid genus Raveniola Zonstein, 1987 (Zonstein 1987), which currently contains 19 species mainly distributed in the Middle East and Central Asia (Platnick 2010). The original description by Brignoli contains some data interpreted erroneously. This species was presented as the largest nemesiid of Eurasia, with a carapace length of up to 10 mm in males and 15 mm in females (actually it was found to be considerably smaller), but the leg measurements of the male holotype given in the same paper were disproportional, amounting to less than half of that necessary to correspond to the stated carapace length (see Brignoli 1972). The original figures showing the configuration of the male palp and the spermathecae of Raveniola niedermeyeri (Brignoli 1972: figs 1–2) are too schematic to permit reliable identification and differentiation from its congeners. These incorrect and incomplete data are corrected in our redescription and new data on the distribution of Raveniola niedermeyeri are provided.

## Material and methods

Specimens from the following institutions were studied: MHNGMuséum d’histoire naturelle, Genève, Switzerland; TAUZoological Museum, Tel Aviv University, Israel; ZMMUZoological Museum of the Moscow State University, Russia.

Other abbreviations are as follows. *Eyes*: ALEanterior lateral, AMEanterior median, PLEposterior lateral, PMEposterior median. *Spinnerets*: PLSposterior lateral, PMSposterior median. *Spine positions*: pprolateral; pdprodorsal; pvproventral; rretrolateral; rdretrodorsal; rvretroventral; vventral.

Photographs were taken either using a Canon 500D digital camera with a 100 mm Canon macro lens and a Zeiss Discovery V20 stereomicroscope with a Canon PowerShot G9 digital camera attached to it. Measurements were taken through a Leica MZ12 stereomicroscope with an accuracy of 0.025 mm (approximated up to the nearest centesimal). All measurements are given in millimetres, except for eye diameters and interdistances which are given in microscope scale units (measured at 100×). Lengths of palps and legs are given as: total (femur, patella, tibia, metatarsus, tarsus).

## Taxonomy

### Raveniola niedermeyeri

(Brignoli, 1972)

Figs 1 8

Brachythele niedermeyeri Brignoli, 1972: 412 (male holotype from Astrabad = now called Gorgan, Iran; deposited in the MHNG, examined); Brignoli 1983: 123; Zonstein 1985: 159.
Raveniola niedermeyeri : Zonstein 1987: 1015; Platnick 1989: 90; Mozaffarian and Marusik 2001: 70; Ghahari and Marusik 2009: 4.

#### Types.

♂ holotype – IRAN: Alborz Mts., surroundings of Gorgan (36°50'N; 54°26'E), date not specified but most probably in 1913–1914, prior to World War I, coll. O.R. Niedermeyer (MHNG). Paratypes: 3♂ 2♀ with the same collecting data (MHNG).

#### Additional material examined.

IRAN: Gorgan, IX.2004, coll. H. Ghahari, 1♂ (TAU); Golestan Province, Aliabad, 36°53'N; 54°57'E, 30.VII.1974, coll. A. Senglet, 1♀, 7 juv. (MHNG); Mazandaran Province, Elborz (Alborz) Mts, oak forest, VI.2004, coll. H. Ghahari, 1♂ 1♀ (ZMMU); Khorasan Province, surroundings of Mashad (36°17'N; 59°36'E), IX.2005, coll. H. Ghahari, 1♀ (TAU); Esfahan Province, surroundings of Esfahan (32°40'N; 51°40'E), XI.2005, coll. H. Ghahari, 1♂ (ZMMU).

**Figures 1–4.**
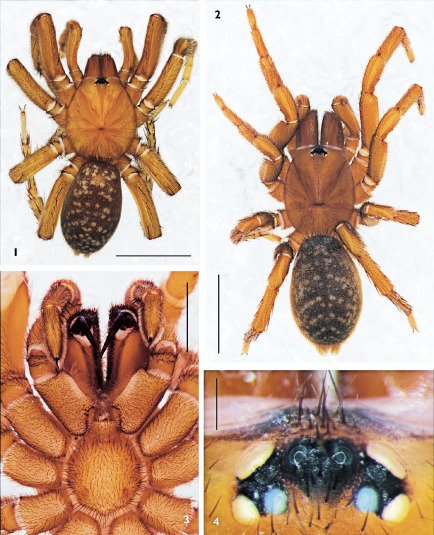
Raveniola niedermeyeri, conspecific male (**1**) and female (**2–4**); **1**, **2** body, dorsal view **3** sternum, labium and maxillae, ventral view **4** eye tubercle, dorsal view. (scale bars: **1**, **2** = 5 mm; **3** = 2 mm; **4** = 0.25 mm).

#### Diagnosis.

The species differs from all other congeners of Raveniola in having a gradually tapering and curved embolus in males (Figs 6, 7), and the lateral receptacles reduced to vestiges in females (Figs 8, 9).

#### Redescription.

Male (holotype). Total body length including chelicerae 13.80. Colour in alcohol: carapace, chelicerae, palps and first pair of legs dorsally intense reddish brown; eye tubercle with darker spots surrounding AMEs and lateral eyes; sternum, labium, maxillae and legs II–IV light reddish brown; abdomen dorsally light greyish brown; typical darker dorsal pattern consisting of a longitudinal median spot crossed by a few poorly preserved transverse fasciae, ventral abdominal surface and spinnerets pale greyish brown.

**Figures 5–9.**
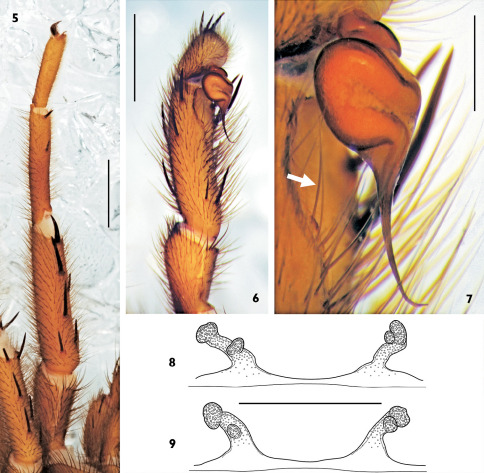
Raveniola niedermeyeri, holotype male (**5–7**) and paratype/conspecific female (**8**, **9**); **5** leg I, patella to tarsus, retrolateral view **6** palp, patella to cymbium, retrolateral view **7** palpal organ, retrolateral view **8**, **9** spermathecae, dorsal view: specimens from Gorgan (paratype) and Mazandaran province, respectively. (scale bars: **5**, **6** = 2 mm; **7**, **8** = 0.5 mm).

General appearance as in Fig. 1. Carapace 5.32 long, 4.55 wide; covered with moderately dense and thin semi-adpressed dark hairs. Eye diameters (AME, ALE, PLE, PME): 14, 26, 18, 16/17. Interdistances: AME–AME 12, ALE–AME 7, ALE–PLE 7, PLE–PME 7/6, PME–PME 33. Cheliceral furrow with 9–10 promarginal teeth and 7–8 mesobasal denticles. Labium 0.42 long, 0.87 wide. Maxillae with 6–7 cuspules. Sternum 2.45 long, 2.28 wide. Palp: 7.62 (2.75, 1.67, 2.23, –, 0.97). Leg I: 14.88 (4.17, 2.67, 3.27, 3.00, 1.77). Leg II: 12.15 (3.70, 2.33, 2.70, 2.67, 1.75). Leg III: 11.97 (3.27, 1.77, 2.25, 3.15, 1.63). Leg IV: 16.27 (4.25, 2.13, 3.33, 4.53, 2.03). Leg I: tibia slightly incrassate, metatarsus slightly curved retroventrally (Fig. 5).

Spination. Palp: femur d1–1–0, pd1, rd1; patella p1–1; tibia d1–1, p1–1–1, r1–1–1, v2–1–1–1; cymbium d4(5). Leg I: femur d1–1–0–0, pd1–1–1; rd 1(0)–1–1(0); patella 0; tibia p1–1–0, v3–2–1–1; metatarsus v1(0)–1. Leg II: femur d1–1–0–0; pd1–1; tibia p1–1–1, v2–2–3; metatarsus p1; v1–2–2–2. Leg III: femur d1–1–0–0, pd0–1–1, rd0–1–1; patella p1–1, r1; tibia d1–1, p1–1–1, r1–1–1, v2–2–2(3); metatarsus d1–1–2, p1–1–1, r1–1–1, v2(3)–2–3. Leg IV: femur d1–1–0–0, pd0–1–1, rd0–1–1; patella p1, r1; tibia d1–1–2, p1–1–1, r1–1–1, v2–2–2(3); metatarsus pd1–1–2, p1–1–1, r1–1–1, v2–1–2–1(0)–3. Patella I and tarsi I–IV aspinose.

Scopula: distally on metatarsus I, entire on tarsus I, divided by setae on tarsus II; elsewhere absent. Paired claws: 8–10 teeth in two rows on each claw. Trichobothria: 2 rows of 8–11 per row on tibiae, 10–13 on metatarsi, 10–12 on tarsi, 8 on cymbium.

Palpal tibia moderately long, provided with ventral subapical sensilla (Figs 6, 7; indicated by arrow in Fig. 7); cymbium spinose. Bulb pyriform with ejaculatory duct sinuous; embolus without keel, gradually tapering and curved ventrad apically (Fig. 7).

Spinnerets. PMS: length 0.25; diameter 0.15. PLS: maximum diameter 0.35; length of basal, medial and apical segments 0.67, 0.55, 0.37; total length 1.59; apical segment triangular.

Female (paratype): Total body length including chelicerae 15.90. Colour in alcohol and pubescence as in male, dorsal abdominal pattern better preserved, consisting of numerous irregularly arranged, small yellowish brown spots on darker brown background.

General appearance, eye tubercle and ventral aspect of sternum, labium and maxillae as in Figs 2, 4 and 3, respectively. Carapace 5.35 long, 4.23 wide. Eye diameters (AME, ALE, PLE, PME): 12, 26, 20, 13. Interdistances: AME–AME 13, ALE–AME 9, ALE–PLE 8, PLE–PME 4, PME–PME 38. Cheliceral furrow with 9 promarginal teeth and 5 mesobasal denticles. Labium 0.54 long, 1.06 wide. Maxillae with 10–11 cuspules. Sternum 2.38 long, 2.30 wide. Palp: 7.48 (2.25, 1.50, 1.73, –, 2.00). I: 11.82 (3.47, 2.30, 2.57, 2.03, 1.45). II: 10.63 (3.05, 2.03, 2.15, 1.93, 1.47). III: 10.32 (2.77, 1.77, 1.80, 2.45, 1.53). IV: 14.54 (3.63, 2.23, 2.92, 3.85, 1.90).

Spination. All femora with 1 basodorsal slender spine and a few stiff bristles (undeveloped spines) located medially and distally; palpal patella, patella I and tarsi I–IV aspinose. Palp: femur d1, pd1; tibia v2–1–2; tarsus d5(6). Leg I: femur d1, pd1; tibia v2–1–2; metatarsus v2–2–2. Leg II: femur d1, pd1; patella p1; tibia p1–1, v2–1–3; metatarsus v2–2–2. Leg III: femur d1, pd 1–1, rd 1–1; patella p1–1, r1; tibia d1, p1–1, r1–1, v2–2–3; metatarsus pd1–1, p1–1–1, r1–1–2, v2–1–3–3. Leg IV: femur d1, rd1; patella p1, r1; tibia p1–1, r1–1–1, v2–2–3; metatarsus d1–1–1, p1–1–1–1, r1–1–1–1, v2–1(2)–2(3)–3.

Scopula: distal on metatarsi I–II, narrowly divided by setae on palpal tarsus and tarsus I, widely divided on tarsus II, elsewhere absent. Paired claws: 5–7 teeth in two rows on each claw, palpal claw with 4 teeth on inner margin. Trichobothria: 2 rows of 7–9 per row on tibiae, 11–14 on metatarsi, 11–14 on tarsi, 9 on palpal tarsus.

Spermathecae as in Figs 8 and 9. Second (lateral) receptacles underdeveloped, sessile, rudimentary to almost absent.

Spinnerets. PMS: length 0.42; diameter 0.20. PLS: maximum diameter 0.55; length of basal, medial and apical segments 0.65, 0.48, 0.40; total length 1.53; apical segment triangular.

#### Variability.

Carapace length in males varies from 5.05 to 5.90; in females from 5.10 to 7.05. The general coloration is usually the same as shown in Figs 1 and 2, with insignificant variation throughout the series of specimens examined. In males, variations in the shape of the bulb and embolus were not evident. Two variants of the female spermathecae, which differ only slightly, are shown in Figs 8 and 9.

#### Distribution.

**Figure 10.**
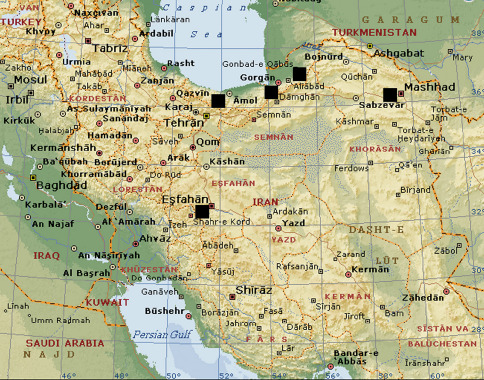
Localities of Raveniola niedermeyeri in Iran.

Iran: Alborz Mts., Khorasan and Zagros Mts. (Fig. 10)

## Supplementary Material

XML Treatment for Raveniola niedermeyeri
